# Test–retest reliability of selected HBSC items in Vietnam: well-being, physical and sedentary activities, and eating behaviours

**DOI:** 10.1186/s12874-022-01624-7

**Published:** 2022-05-12

**Authors:** Jaroslav Kohoutek, Marek Maráček, Kwok Ng, Zdenek Hamrik

**Affiliations:** 1 Department of Recreation and Leisure Studies, Faculty of Physical Culture, Palacký University Olomouc, 77147 Olomouc, Czech Republic; 2 Physical Activity for Health Research Cluster, Department of Physical Education and Sport Sciences, University of Limerick, Limerick, V94 T9PX Ireland; 3 School of Educational Sciences and Psychology, University of Eastern Finland, Joensuu, 80101 Finland

**Keywords:** HBSC, Measures, Adolescent’s, Screen-time, Surveys

## Abstract

**Background:**

Valid and reliable research tools to assess children’s and adolescent’s health-related behaviour are highly needed across the globe. Rapid economic development, globalization, and associated lifestyle challenges observed in most countries support the need for high-quality evidence in adolescents to target health-promoting policies and interventions. This study aims to examine the test–retest reliability of selected well-being, physical and screen-time related siting activities, and eating behaviour items of the Health Behaviour in School-Aged Children (HBSC) questionnaire in a sample of Vietnamese adolescents.

**Methods:**

Data were collected in autumn 2018 in Vietnam (3-week interval). The sample consisted of 410 adolescents (41.0% of boys; mean age = 12.61; SD = 1.24).Test–retest reliability was evaluated using the single measure Intraclass Correlation Coefficients (ICC) and Cohen’s kappa statistic stratified by sex, grade and place of residence (urban or rural).

**Results:**

The reliability analyses of the well-being items were poor to good ICC values (0.43–0.79) and moderate to large Cohen’s kappa values (0.33–0.77). The physical activity and eating behaviour items were moderate (ICC = 0.54–0.65; Cohen’s kappa = 0.38–0.57). The screen-time related siting activities items were moderate to large (ICC = 0.51–0.72; Cohen’s kappa = 0.42–0.53). There was more item stability among females than males. The social media item was not as stable for 6^th^ graders (ICC = 0.45) compared with older adolescents (ICC 0.68–0.77).

**Conclusions:**

The findings show that with regards to age, sex and place of residence, self-reported health, life satisfaction, physical and screen-time related siting activities, as well as eating behaviour items of the HBSC questionnaire have a sufficient test–retest reliability to be used in national self-report surveys for Vietnamese adolescents while health complaints items showed borderline reliability.

## Background

Children’s health and health behaviours are one of the global public health issues. Health-related habits that consolidate in adolescence subsequently persist into adult wellbeing, development of health complaints, tobacco use, diet, physical activity levels, and alcohol use [1, 2]. Thus, it is of high of importance to use valid and reliable research instruments to assess children’s and adolescent’s health and health-related behaviour.

One of the largest international surveys on adolescent health and health-related behaviours is the WHO collaborative Health Behaviour in School-Aged Children (HBSC) study which focuses on 11-, 13- and 15-year-old boys’ and girls’ health and well-being in their social context (school, family, and friends) [3]. The HBSC questionnaire is currently used in 50 countries, mostly in Europe and North American [4]. To date, the HBSC questionnaire has been used within linked projects in Australia, Brazil, Chile, China, Hong Kong, Lebanon, Mozambique, Sao Tome and Principe, Saudi Arabia and Taiwan. Thus, there is a strong need to examine the validity and reliability of the survey instrument in different continents and cultures [5].

In the past 30 years, Vietnamese economy has rapidly grown, and Vietnam which had been recognized as one of the world’s poorest countries is now considered a low-middle income country. Since the 1990s, 30 million people have exceeded the official poverty line, and until 2015, GDP per citizen increased from $100 to $2,300 [6]. The GDP growth rate in 2018 was the highest since 2008 [7]. Despite these developments, there is significant economic inequality affecting opportunity, health, and education [6, 8]. The population of Vietnam was 94.7 million people (49.4% male) in 2018. There are 16.5 million pupils in general schools of whom 5.4 million are in Vietnamese lower secondary schools (aged 11–15). Approximately a third of lower secondary school pupils are in urban areas (1.8 million) while the remaining pupils are in rural areas (3.6 million) [8].

The rapid socio-economic development has brought new challenges in lifestyle and health behaviour. Weiss et al. [9] reported that approximately 12% of Vietnamese children experienced significant mental health problems. In 2013, one in five (20%) lower secondary school pupils in Ho Chi Minh City was classified as overweight with a further 8% with obesity [10, 11]. Child obesity continues to increase [12] at the same rate in which more families have improved economic situations [10, 13]. Vietnamese families with a higher socioeconomic status can also provide their children with a modern lifestyle, such as computers, the internet, and television, which causes these children to be more physically inactive by increased leisure time spent by screen-time activities [14]. Overall, the majority of adolescents in Ho Chi Minh City spent >  = 2 h/day in screen-time activities which are also highly associated with the increase in their overweight and obesity prevalence (21%) [15]. Eating behaviour has also changed over the last decade which contributes to childhood overweight and obesity [16–18]. The replacement of the traditional diet based on cereal, tuber, and vegetables expanded to include meat, eggs, milk, fat, and sugar. Fast foods and drinks, animal-based foods, and refined carbohydrates (sugars, sweets) represent the most significant phenomenon of the nutritional transition [16].

In this context, the importance of valid and reliable research tools for adolescents is highly relevant. Although some test–retest studies of selected HBSC questionnaire items have been carried out [19–24], the number of studies assessing the reliability of research instruments in Asia conditions is still limited [5]. Thus, the aim of the present study was to examine the test–retest reliability of selected well-being, physical and screen-time related siting activities, and eating behaviour items of the HBSC questionnaire in a sample of Vietnamese adolescents.

## Methods

### Sample and procedure

The data for this test–retest study were collected between November and December 2018 in Vietnam. The study is based on the HBSC study and its methodology [25].

Four elementary schools located in rural as well as urban areas in the Hồ Chí Minh City, Đồng Nai Province, and Tiền Giang Province were randomly selected for the study. Trained researcher assistants administrated the questionnaires during regular class time in the 6^th^, 7^th^, 8^th^, and 9^th^ grades (615 registered pupils) without the presence of the teacher.

In the first part of the study (Test), we collected data from 525 adolescents (response rate 85.37%). Non-participation of in this study was due to illness (2.76%) and because of other reasons that the research team were not able to identify (11.87%). The second part of the study (Retest) was conducted 3 weeks after the test. In the retest, we collected data from 504 adolescents (response rate: 81.95%). 19 adolescents who had participated in the first part of data collection (Test) dropped out. 94 adolescents could not be paired by ID codes. The final sample consisted of 410 adolescents (boys 40.2%) and was stratified by grade, sex, and place of residence (Table 1).

**Table 1:** Demographic characteristics of sample

	**Class**		**Sex**				**Residence**				**Age**	
	**n**	**%**	**Boy**		**Girl**		**Urban**		**Rural**		**Mean age**	**Standard Deviation**
			**n**	**%**	**n**	**%**	**n**	**%**	**n**	**%**		
6^th^ grade	77	18.8	33	42.9	44	57.1	39	50.6	38	49.4	10.9	0.44
7^th^ grade	110	26.8	45	40.9	65	59.1	60	54.5	50	45.5	11.9	0.40
8^th^ grade	115	28.0	38	33.0	77	67.0	59	51.3	56	48.7	12.9	0.36
9^th^ grade	108	26.4	49	45.4	59	54.6	64	59.3	44	40.7	14.2	0.60
Total	410	100	165	40.2	245	59.8	222	54.1	188	45.9	12.6	1.24

### Questionnaire items

All items were part of the 2018 HBSC questionnaire and had been previously tested in HBSC countries [19, 23]. Before the start of the study, the translation and back-translation of the questions was conducted, as well as a focus group with students to ensure the comprehensibility and understanding of the questions.

Self-rated health was assessed using the question: “Would you say your health is…?” Possible responses included: Excellent; Good; Fair or Poor. The cut-off point for self-rated health was excellent [4, 26].

Life satisfaction was measured by the question: “In general, where on the ladder do you feel you stand at the moment? Tick the box next to the number that best describes where you stand.” Responses ranged on a scale from 0 to 10. The question was preceded by the explanatory text: “Here is a picture of a ladder. The top of the ladder “10” is the best possible life for you and the bottom “0” is the worst possible life for you.” Higher scores indicate a greater level of perceived satisfaction. The cut-off point for the dichotomization of life satisfaction was 8 and more [4, 27].

Individual health complaints (headache, stomach ache, backache, feeling low, feeling irritable, feeling nervous, sleep difficulties, and feeling dizzy) were measured by asking a question for each individual health complaint: “In the last 6 months: how often have you had the following….?” Please tick one box for each line”. Responses were rated on a five-point scale: About every day; More than once a week; About every week; About every month; Rarely or never. The cut-off point the for dichotomization of individual health complaints was more than once a week [4].

Moderate to vigorous physical activity (MVPA) was measured by the question: “Over the past 7 days, on how many days were you physically active for a total of at least 60 min per day? Please add up all the time you spent in physical activity each day.” Possible responses were selected on a scale from 0 to 7 days. The question was explained by text: “Physical activity is any activity that increases your heart rate and makes you get out of breath some of the time. Physical activity can be done in sports, school activities, playing with friends, or walking to school. Some examples of physical activity are running, brisk walking, rollerblading, biking, dancing, skateboarding, swimming, soccer, basketball, football, & surfing”. The cut-off point for the dichotomization of MVPA was 60-min MVPA 7 days [4, 21, 28].

Vigorous physical activity (VPA) was measured by asking the question: “Outside school hours: how often do you usually exercise in your free time so much that you get out of breath or sweat?” The following multiple-choice answers were offered: Every day; 4–6 times a week; 2-–3 times a week; Once a week; Once a month; Less than once a month; Never. The cut-off point for VPA was 4 or more times a week [4].

Screen-time related siting items were evaluated by asking the question for each item: “In your leisure time, how many hours a day do you spend.…: a) … playing games on a computer, game console, tablet, smartphone, or smart TV?; b) … using a computer or another electronic device (for example smartphone or tablet) for a different purpose (for example, social and communication networks – Instagram, Twitter, Snapchat, Facebook, etc., chatting or surfing the internet)?; c) … watching internet videos (for example on YouTube or Twitch)?; d) … watching TV, DVDs or videos (do not include internet videos on websites such as YouTube)?; Please tick one box in each row.” Possible multiple-choice responses: Not at all; About half an hour a day; About 1 h a day; About 2 h a day; About 3 h a day; About 4 h a day; About 5 h a day; About 6 h a day; About 7 or more hours a day. The cut-off point for screen-time related siting items (gaming, social media, internet videos, TV or DVDs) was 2 h a day [4, 19, 29, 30].

Breakfast consumption on school days was assessed using the question: “How often do you usually have breakfast (more than a glass of milk or fruit juice)?” Response options included: Never; One day; Two days; Three days; Four days; Five days. The cut-off point for breakfast consumption on school days was every school day [4].

Eating behaviour (Fruit, vegetables, sweets, and sugared soft drinks consumption) was evaluated using the question for each item: “How many times a week do you consume….? Please tick one box in each row.” Possible response options were: Never; Less than once a week; Two to four times a week; Five to six times a week; Once a day; More than once a day. The cut-off point for eating behaviour items (Fruit, vegetables, sweets, and sugared soft drinks consumption) was daily [4].

### Statistical analyses

Descriptive statistics and the Chi-square test were used to characterize the responses in the test and retest (Table 2). To assess the test–retest reliability of the selected items, the single measure of the Intraclass Correlation Coefficient (ICC) was used. ICCs were computed from a two-way mixed-effects ANOVA model for absolute agreement of single measures [31]. All of the selected items were stratified by class, sex, residence. The strength of the test–retest agreement was evaluated according to Koo and Li [31]: Larger than 0.90 indicates excellent agreement; 0.75–0.9 indicates good agreement; 0.5–0.75 represents moderate agreement and below 0.50 is classified as poor agreement.

**Table 2:** Rates of dichotomous health behaviour responses and means during Test–Retest

	Test (*n* = 410)	Mean	SD	Retest (*n* = 410)	Mean	SD	*p* (%)
	%			%			
**Health**							
Self-rated health	48.0	1.67	0.73	46.1	1.70	0.75	0.71
Life satisfaction	52.5	7.37	1.82	54.6	7.38	1.86	0.67
**Health complaints**							
Headache	16.8	4.24	1.28	16.2	4.26	1.22	0.93
Stomach ache	10.6	4.47	1.07	9.5	4.51	0.99	0.87
Backache	11.8	4.42	1.11	12.6	4.32	1.18	0.91
Feeling low	44.4	3.07	1.60	44.4	3.04	1.52	1
Irritability	39.8	3.32	1.60	35.4	3.36	1.51	0.44
Nervous	36.8	3.36	1.58	38.9	3.35	1.55	0.71
Difficult sleep	16.8	4.27	1.30	19.9	4.14	1.36	0.63
Feeling dizzy	14.1	4.35	1.19	14.2	4.35	1.17	0.99
**Physical activity**							
MVPA	14.5	3.18	2.21	15.6	3.06	2.32	0.87
VPA	22.1	3.82	1.79	22.9	3.97	1.90	0.90
**Screen-time**							
Gaming	62.5	3.23	1.88	61.0	3.28	1.94	0.73
Social media	67.2	3.20	2.06	69.2	3.18	2.08	0.61
Internet videos	57.6	3.69	1.88	55.4	3.66	1.90	0.63
TV, DVD	71.1	2.93	1.91	71.0	2.85	1.77	0.98
**Eating behaviour**							
Breakfast	62.5	4.91	1.68	62.3	4.96	1.60	0.96
Fruit	38.8	4.80	1.68	36.2	4.61	1.74	0.64
Vegetables	50.0	5.03	1.88	53.0	5.11	1.75	0.54
Sweets	13.3	3.35	1.73	16.2	3.40	1.72	0.66
Soft-Drinks	19.4	3.75	1.76	21.0	3.76	1.77	0.80

% represents the percentage responses rates of dichotomous items; mean represents the response value on whole-scale of items; *p*—value based on chi-square test

All of the selected items were dichotomized and cut-off points for the dichotomization were set according to the international HBSC report, “Spotlight on adolescent health and well-being” [32]. Then, the Cohen’s simple Kappa statistic was used to measure the agreement between binary variables. According to Cohen [33] the Cohen’s Kappa correlation is classified as: greater than 0.5 indicates large agreement; 0.3–0.5 indicates moderate agreement, 0.1–0.3 small agreement and less than 0.1 is classified as trivial. For all statistical analyses, the IBM SPSS 20 for Windows was used.

## Results

**Fig. 1:**
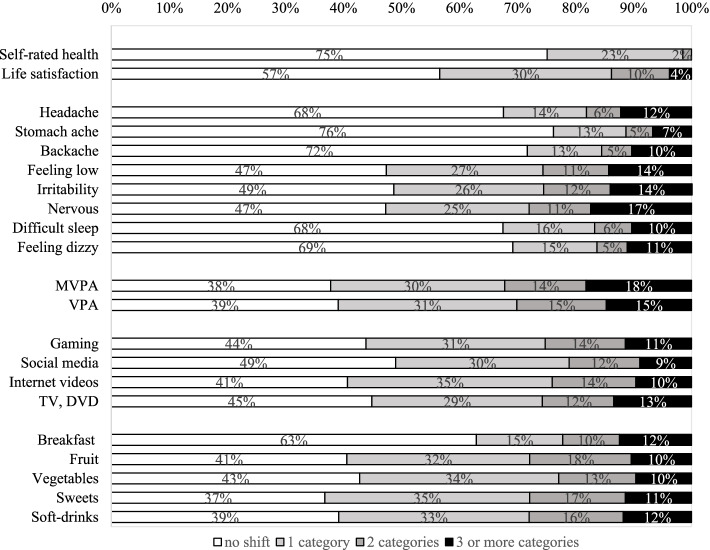
Percentages of test–retest response shifts on selected items of HBSC questionnaire

In the final sample of 410 Vietnamese adolescents (mean age = 12.6, SD = 1.2), there were more girls (60%) than boys (40%) and slightly more respondents were from urban areas (54%) than rural areas (46%). The differences in the rates of each of the health behaviour item cut-offs were not statistically significant (Table 2), proportions of no response shift between test and retest varied from 37 to 75% (Fig. 1), implying good stability of items among Vietnamese adolescents. The highest rate was watching TV or DVDs for 2 or more hours (71%) while the lowest rate was stomach ache more than once a week (10–11%).

Based on the total sample, there was poor to good agreement on the use of the full scale measured by ICC (Table 3) and moderate to large agreement for binary variables (Table 4).

**Table 3:** ICC for selected items of HBSC questionnaire by sex grade and residence

Variables	ALL (*n* = 410)		Boys (*n* = 165)		Girls (*n* = 245)		6 (*n* = 77)		7 (*n* = 110)		8 (*n* = 115)		9 (*n* = 108)		Urban (*n* = 222)		Rural (*n* = 188)	
	**ICC**	**95% CI**	**ICC**	**95% CI**	**ICC**	**95% CI**	**ICC**	**95% CI**	**ICC**	**95% CI**	**ICC**	**95% CI**	**ICC**	**95% CI**	**ICC**	**95% CI**	**ICC**	**95% CI**
**Health**																		
Self-Rated Health	0.73	0.68–0.77	0.65	0.55–0.73	0.78	0.72–0.82	0.82	0.74–0.88	0.73	0.63–0.81	0.71	0.60–0.79	0.68	0.56–0.77	0.72	0.66–0.78	0.74	0.67–0.80
Life Satisfaction	0.79	0.75–0.83	0.80	0.73–0.85	0.79	0.73–0.83	0.77	0.65–0.85	0.81	0.72–0.87	0.85	0.79–0.89	0.73	0.63–0.81	0.82	0.76–0.86	0.76	0.68–0.81
**Health complaints**																		
Headache	0.48	0.40–0.55	0.43	0.29–0.55	0.51	0.40–0.60	0.43	0.23–0.60	0.59	0.45–0.71	0.57	0.43–0.69	0.32	0.14–0.48	0.51	0.41–0.61	0.44	0.31–0.55
Stomach ache	0.56	0.48–0.62	0.61	0.50–0.70	0.52	0.42–0.61	0.64	0.48–0.77	0.37	0.19–0.54	0.61	0.48–0.72	0.57	0.43–0.70	0.59	0.49–0.67	0.51	0.39–0.61
Backache	0.46	0.37–0.54	0.45	0.31–0.57	0.46	0.35–0.56	0.41	0.18–0.59	0.50	0.34–0.64	0.55	0.40–0.67	0.35	0.17–0.51	0.47	0.36–0.57	0.44	0.30–0.55
Feeling low	0.53	0.45–0.60	0.54	0.41–0.64	0.51	0.41–0.60	0.39	0.17–0.57	0.61	0.46–0.72	0.52	0.36–0.64	0.52	0.36–0.65	0.60	0.51–0.68	0.44	0.31–0.55
Irritability	0.51	0.43–0.58	0.50	0.36–0.61	0.51	0.41–0.60	0.36	0.13–0.55	0.49	0.32–0.63	0.52	0.37–0.65	0.62	0.49–0.73	0.53	0.42–0.62	0.48	0.36–0.59
Nervous	0.43	0.35–0.51	0.39	0.24–0.51	0.46	0.35–0.55	0.52	0.33–0.67	0.40	0.22–0.56	0.30	0.11–0.46	0.51	0.35–0.64	0.50	0.39–0.60	0.34	0.20–0.46
Sleep difficulties	0.57	0.50–0.63	0.44	0.31–0.56	0.65	0.56–0.71	0.46	0.24–0.63	0.48	0.32–0.62	0.70	0.59–0.79	0.59	0.45–0.70	0.56	0.46–0.65	0.55	0.43–0.65
Feeling dizzy	0.47	0.39–0.55	0.24	0.08–0.39	0.57	0.48–0.65	0.51	0.32–0.66	0.37	0.18–0.52	0.57	0.42–0.69	0.43	0.26–0.57	0.42	0.30–0.52	0.55	0.43–0.64
**Physical activity**																		
MVPA	0.58	0.51–0.64	0.55	0.44–0.65	0.54	0.44–0.62	0.63	0.48–0.80	0.68	0.56–0.77	0.60	0.43–0.68	0.45	0.29–0.60	0.49	0.38–0.59	0.67	0.58–0.74
VPA	0.57	0.50–0.63	0.49	0.36–0.60	0.51	0.41–0.60	0.55	0.37–0.69	0.48	0.32–0.61	0.61	0.47–0.71	0.64	0.51–0.74	0.52	0.42–0.61	0.61	0.52–0.70
**Screen-time**																		
Gaming	0.62	0.55–0.68	0.60	0.50–0.69	0.63	0.54–0.70	0.62	0.46–0.74	0.67	0.56–0.77	0.52	0.37–0.64	0.64	0.51–0.74	0.65	0.57–0.72	0.55	0.44–0.64
Social media	0.72	0.67–0.77	0.57	0.45–0.66	0.77	0.71–0.81	0.45	0.25–0.61	0.73	0.63–0.81	0.68	0.57–0.77	0.77	0.68–0.84	0.73	0.66–0.79	0.71	0.63–0.77
Internet videos	0.67	0.61–0.72	0.55	0.44–0.65	0.72	0.65–0.78	0.72	0.59–0.81	0.67	0.56–0.77	0.65	0.53–0.75	0.61	0.48–0.72	0.63	0.54–0.70	0.71	0.63–0.77
TV, DVD	0.51	0.44–0.58	0.58	0.46–0.67	0.47	0.37–0.56	0.69	0.55–0.79	0.34	0.16–0.49	0.49	0.34–0.62	0.63	0.50–0.73	0.56	0.50–0.64	0.45	0.33–0.56
**Eating behaviour**																		
Breakfast	0.54	0.46–0.61	0.40	0.26–0.56	0.61	0.53–0.69	0.45	0.25–0.61	0.50	0.34–0.64	0.65	0.52–0.74	0.55	0.40–0.67	0.57	0.47–0.65	0.50	0.38–0.60
Fruit	0.56	0.49–0.62	0.51	0.39–0.62	0.60	0.51–0.67	0.52	0.34–0.67	0.53	0.38–0.65	0.60	0.47–0.70	0.58	0.43–0.70	0.56	0.46–0.64	0.56	0.46–0.66
Vegetable	0.65	0.60–0.70	0.59	0.47–0.68	0.70	0.63–0.76	0.57	0.40–0.70	0.66	0.54–0.76	0.76	0.67–0.83	0.60	0.46–0.71	0.64	0.55–0.71	0.68	0.59–0.75
Sweets	0.57	0.50–0.63	0.42	0.28–0.54	0.65	0.56–0.71	0.40	0.18–0.57	0.58	0.44–0.69	0.62	0.49–0.72	0.57	0.42–0.68	0.53	0.42–0.61	0.59	0.49–0.68
Soft-Drinks	0.60	0.53–0.66	0.55	0.44–0.65	0.63	0.54–0.70	0.50	0.31–0.65	0.62	0.49–0.72	0.72	0.62–0.80	0.47	0.31–0.61	0.57	0.47–0.66	0.58	0.47–0.66

**Table 4:** Cohen’s kappa for selected items of HBSC questionnaire by sex, grade and residence

Variables	ALL (*n* = 410)	Boys (*n* = 165)	Girls (*n* = 245)	6 (*n* = 77)	7 (*n* = 110)	8 (*n* = 115)	9 (*n* = 108)	Urban (*n* = 222)	Rural (*n* = 188)
	**Cohen’s Kappa**	**Cohen’s Kappa**	**Cohen’s Kappa**	**Cohen’s Kappa**	**Cohen’s Kappa**	**Cohen’s Kappa**	**Cohen’s Kappa**	**Cohen’s Kappa**	**Cohen’s Kappa**
**Health**									
Self-Rated Health	0.67**	0.56**	0.73**	0.73**	0.65**	0.61**	0.66**	0.63**	0.71**
Life Satisfaction	0.77**	0.70**	0.81**	0.79**	0.80**	0.76**	0.72**	0.80**	0.73**
**Health complaints**									
Headache	0.35**	0.37**	0.33**	0.36*	0.51**	0.31*	0.23*	0.40**	0.29**
Stomach ache	0.47**	0.58**	0.41**	0.58**	0.49**	0.53**	0.34**	0.51**	0.42**
Backache	0.35**	0.37**	0.34**	0.29*	0.39**	0.37**	0.34**	0.42**	0.25*
Feeling low	0.40**	0.45**	0.37**	0.35*	0.50**	0.37**	0.36**	0.43**	0.37**
Irritability	0.40**	0.41**	0.39**	0.26	0.39**	0.44**	0.44**	0.41**	0.40**
Nervous	0.35**	0.24*	0.40**	0.37*	0.34*	0.29*	0.39**	0.41**	0.27**
Sleep difficulties	0.43**	0.34**	0.48**	0.36*	0.37**	0.51**	0.47**	0.42**	0.42**
Feeling dizzy	0.33**	0.10	0.42**	0.42**	0.25*	0.33*	0.34**	0.28**	0.40**
**Physical activity**									
MVPA	0.46**	0.46**	0.37**	0.31*	0.53**	0.65**	0.36**	0.37**	0.55**
VPA	0.42**	0.43**	0.22*	0.31*	0.43**	0.31*	0.51**	0.30**	0.56**
**Screen-time**									
Gaming	0.50**	0.48**	0.51**	0.67**	0.46**	0.45**	0.49**	0.51**	0.47**
Social media	0.53**	0.41**	0.55**	0.31*	0.45**	0.46**	0.64**	0.53**	0.53**
Internet Videos	0.52**	0.46**	0.53**	0.53**	0.48**	0.52**	0.50**	0.45**	0.60**
TV, DVD	0.42**	0.49**	0.38**	0.49**	0.50**	0.31*	0.44**	0.39**	0.46**
**Eating behaviour**									
Breakfast	0.57**	0.57**	0.57**	0.42**	0.52**	0.65**	0.64**	0.63**	0.50**
Fruit	0.46**	0.39**	0.50**	0.41**	0.46**	0.40**	0.53**	0.46**	0.46**
Vegetable	0.50**	0.46**	0.53**	0.48**	0.54**	0.53**	0.43**	0.55**	0.44**
Sweets	0.38**	0.22*	0.44**	-0.05	0.45**	0.35**	0.39**	0.29**	0.44**
Soft-Drinks	0.43**	0.35**	0.48**	0.26*	0.46**	0.52**	0.37**	0.45**	0.39**

^*^p < 0.05, **p < 0.001

The ICCs of self-rated health and life satisfaction items ranged from 0.65 to 0.85, depending on sex, age, or place of residence. After testing for changes based on the cut-off values, there was also large agreement with kappa ranging from 0.56 to 0.81, depending on sex, age and place of residence.

There was poor to moderate agreement in the health complaint items with ICCs ranging from 0.24 (boys feeling dizzy) to 0.70 (8^th^ graders experiencing sleep difficulties) after stratifying by sex, age and place of residence (Table 3). The Kappas for the dichotomized items ranged from large to almost trivial (Table 4). For example, items with large agreement were stomach ache for boys (k = 0.58), 6^th^ graders (k = 0.58), 8^th^ graders (k = 0.53) and urban residents (k = 0.51). After dichotomizing the item for feeling dizzy, there was trivial to small agreement among boys (k = 0.1), yet there was moderate agreement among girls (k = 0.42). Furthermore, there was small agreement for 7^th^ graders (k = 0.25) and urban residents (k = 0.28) for the feeling dizzy item.

There was also poor to moderate agreement in physical activity items as a scale (ICC = 0.45–0.68) depending on sex, age or place of residence. When treating the items as dichotomous variables, there was moderate (boys, girls, 6^th^, 9^th^ graders, and urban) to large agreement (7^th^, 8^th^ graders, and rural) for MVPA. Using the cut-off for at least 4 times a week of vigorous exercise, there was small agreement for girls (k = 0.22), large agreement for 9^th^ graders (k = 0.51) and rural residents (k = 0.56), as well as moderate agreement for boys, other age groups and urban residents.

Depending on sex, age, or place of residence, in screen-time items there was poor to good agreement (ICC = 0.45–0.77), with the exception of 7^th^ graders reporting TV or DVD time (ICC = 0.34). 

Based on the cut-off for 2 h per day per purpose, the stability was greater with moderate to large agreement depending on sex, age or place of residence.

There was poor agreement in breakfast consumption for boys (ICC = 0.40) and for 6^th^ graders (ICC = 0.45), and also in sweets consumption for boys (ICC = 0.42) and for 6^th^ graders (ICC = 0.40). Otherwise, there was moderate to good agreement for other sex, age, or place of residence analyses in eating behaviours, with eating vegetables (ICC = 0.57–0.76) being the most stable eating behaviour across the different strata. There was small agreement in consuming sweets daily among boys (k = 0.22) and urban residents (k = 0.29) as well as 6^th^ graders reporting daily soft drink consumption (k = 0.26). The other daily food consumptions were interpreted as having moderate to substantial test–retest agreement (k = 0.35–0.65) depending on sex, age, or place of residence.

## Discussion

The present study provided a snapshot of health behaviours among Vietnamese adolescents and examined the test–retest reliability of selected well-being, physical and sedentary activity, as well as eating behaviour items of the Health Behaviour in School-Aged Children (HBSC) survey. If these results were representative of Vietnamese adolescents and were compared with the other countries in the HBSC study [32], Vietnamese adolescents would have the highest rates of daily feeling low (44%) and the lowest rates of vigorous exercise on at least 4 days a week (22–23%). Considering the majority of the items dealing with self-rated health, life satisfaction, physical activity, screen time and eating behaviour being interpreted to have poor to good agreement, there is confidence that these items are sufficiently reliable for use in Vietnamese conditions for population-based studies. The health complaints items seem to have borderline reliability and their use should be further researched alongside the reasons for the low demonstrated reliability. Our results also indicate the stability of the items on the population level, while shifts in responses could be seen on the individual level. To our knowledge, this is the first study assessing test–retest reliability of well-being, physical and sedentary activity, as well as eating behaviour related items that are commonly used in large population-based studies such as the HBSC or Global School-Based Student Health Survey (GSHS) in the Vietnamese environment.

Similarly to earlier studies that tested these measures in different contexts such as China [5] and central Europe [19], the findings from this study continue to demonstrate the versatility of some measures in different cultural contexts. In some cases, the low ICC and Cohen’s Kappa were reported. The reasons for this might be that the stratification yielded a low number of responses per category which indicates the necessity to plan future studies that can detect sufficient counts of low incidences of certain cut-off values or limit the choice of statistical methods. Secondly, the low ICC and Cohen’s Kappa also indicates that in a specific set of questions, cultural context plays an important role and the use of such questions should be carefully considered. We believe this to be another important result of our study whereas the number of self-reported studies in Asia is growing, similar sets of questions are commonly used in other population-based studies and self-reported measures are often the only feasible method for the measurement of health behaviour in developing countries [34]. On the other hand, our study followed the Koo & Li [31] guideline for reporting ICC while many other studies [5, 19, 20, 23] used milder criteria. In this context, it should be pointed out that other significant studies in the field also recommend even stricter criteria [35].

The results from this study concerning the instruments such as self-rated health and life satisfaction were similar to previous studies in the field [36, 37]. The good to large level of agreement for the self-rated health item extends the existing knowledge of it being a valid and reliable measure of physical and mental health across 19 countries in Europe [38] to the Vietnamese adolescent population. Moreover, the Cantril ladder used to measure life satisfaction in the adolescent population was tested as valid and reliable [36], seems to be stable over time [39] and shows very good repeatability reliability with Pearson correlations with *r* = 0.70, *p* < 0.001 [37].

The reliability of the health complaint items seems to be the lowest in our study. This is in contrast to a previous study conducted in Norway [22] in which the ICC coefficient showed substantial to almost perfect (ICC = 0.61–0.81) agreement. There was more stability across the items among girls than boys for both the full-scale response and the use of cut-off values, particularly for the nervous and feeling dizzy items. As the scores were the lowest, it should be questioned whether answering the set of questions on health complaints might be culturally determined or inappropriate in some countries which may reduce children’s willingness to answer consistently. The Vietnamese culture has been influenced by the Confucian tradition characterized by affective control (group-disturbing emotions such as anger), harmony, and self-control in interpersonal relationships [9, 40]. This might suppress certain child behaviours (e.g., aggression) and support others (through modelling and empowerment), with the result that the relative levels of different types of child psychopathology vary across cultures [9, 41, 42].

Similar results concerning the measures of MVPA and VPA were also reported by other studies based in European countries such as the Czech Republic, Slovakia, Poland (ICC = 0.60–0.62) [19] or Finland (ICC = 0.69–0.77) [43]. Moreover, improved agreement was reported by other studies across the globe with ICC ≥ 0.72 [5, 21, 44]. Ng et al. [43] highlighted that the test–retest period for the MVPA item should be considered when comparing results from different studies. Shorter periods between test-retests can cause respondents to remember their previous answers which may result in higher reliability, but it also depends on the cognitive load between testing. It is also necessary to take sociocultural differences between Europe and Asia into account. These differences may increase or decrease item reliability in some countries. It might be important to know the number of physical education classes per week and other culturally determined facts that could affect physical activity levels and thus the answering of repeated questions in the test–retest procedure. Also, the seasonable character of some sports activities, sociocultural issues, current morbidity (seasonal epidemics), and other specific circumstances, such as different levels of demands placed on students during the school year, might influence the results. Countries with a more stable climate and weather could note greater agreement in any season as this element is eliminated and does not interfere with the results.

Overall, screen time measures showed poor to good ICC and moderate to large (Kappa) agreement for the test–retest reliability. Similar results were reported by another study based on adolescents in the Central European region [19]. In other contexts, Liu et al. [5] examined the reliability of similar screen-time-related sitting items in Asia on a sample of 91 Chinese school-aged children and reported a wider variance in the results. As new technologies keep on evolving and spreading quickly (smartphones, tablets, etc.), we modified and restructured the question to a single set of questions and focused only on weekdays to minimize the suspected overestimation of the previously used HBSC screen-time related sitting questions.

The reliability of eating behaviour items reached moderate to good test–retest agreement in most of the categories. The questions on eating behaviours have been previously recognized as simple and easy to understand in the adolescent population [24]. Most of the studies in the field across the countries in the HBSC network reported similar results. The responses from adolescents in European countries showed very good repeatability reliability [24, 45, 46]. A study from New Zealand [47] showed good to excellent reliability and reasonable validity. Speck, Bradley, Harell, & Belyea [48] who used a 24-h diet recall for assessing the validity of the questionnaire reported confidence in the reliability and validity of the measures to assess the majority of adolescent food intakes. Thus, we assume that eating-related questions have substantial reliability in the identification of the basic dietary habits such as having breakfast, fruit and vegetables, sugar-sweetened beverages and sweet consumptions among adolescents in Vietnam. These eating habits can be further associated with non-communicable diseases such as obesity, cardiovascular disease, cancer, and diabetes [49].

### Strengths and limitations

To our best knowledge, this is the first study assessing the test–retest reliability of well-being, physical and screen-time related siting activities, as well as eating behaviour related items among adolescents in Vietnam. However, there are also some limitations of the study to be mentioned. This test–retest reliability study evaluates only the stability of the questions over time and does not investigate the construct validity of the measures. Although there are some ways to evaluate against a criterion from other clinical surveys, other health behaviours may be more complex and may require a separate study, for example device-based measurements of physical activity behaviour.

The sample contained 40.2% of boys and 59.8% of girls, although the average proportion of female students in lower secondary level is around 48.5% [50]. The reason for this is the higher rate of incomplete questionnaires which were not paired in the test/retest survey round as well as the higher rate of absence of boys.

When taking into account the economic status and cultural background, it seems to be challenging to interpret the results without a diverse sample in Asian countries. This can undermine representativeness and population generalizability. Thus the representativeness and generalizability of our results should be carefully considered. Also, the results should not be generalized to all Asian cultures.

Although a three-week interval was used before the administration of the retest, which seems to be sufficiently long to avoid the retention of previously chosen answers, it can also be a limitation. Some changes may have occurred in the behaviour of some participants between the test–retest administration period. Another limitation is that screen-time related siting activities can be pursued at the same time (e.g., social media while watching TV). However, the items presented in the study do not take this into account.

## Conclusions

The self-reported health, life satisfaction, physical and screen-time related siting activities, as well as eating behaviour items from the HBSC study were seen to have acceptable test–retest reliability test to be used among Vietnamese adolescents. The health complaints items showed borderline reliability; its use should be further researched and the socio-cultural context should be carefully considered. As the HBSC study aims to understand adolescent health and health behaviours in a cross-national way as well as within countries, data collection in Vietnam is desirable. The outcomes of such work could shed light on further health inequalities and allow for the monitoring of the sustainable development goals agenda.

## Data Availability

The data sets used and analysed in the current study are available from the corresponding author on reasonable request.

## References

[1] Due P, Krølner R, Rasmussen M, Andersen A, TrabDamsgaard M, Graham H, Holstein BE (2011). Pathways and mechanisms in adolescence contribute to adult health inequalities. Scand J Public Health.

[2] Sawyer SM, Afifi RA, Bearinger LH, Blakemore SJ, Dick B, Ezeh AC, Patton G (2012). C: Adolescence: a foundation for future health. Lancet.

[3] Currie C, Gabhainn SN, Godeau E (2009). The health behaviour in school-aged children: WHO collaborative cross-national (HBSC) study: origins, concept, history and development 1982–2008. Int J Public Health.

[4] Inchley J, Currie D, Cosma A, Samdal O (2018). Health Behaviour in School-aged Children (HBSC) study protocol: Background, methodology and mandatory items for the 2017/18 survey.

[5] Liu Y, Wang M, Tynjälä J, Lv Y, Villberg J, Zhang Z, Kannas L (2010). Test-retest reliability of selected items of Health Behaviour in School-aged Children (HBSC) survey questionnaire in Beijing, China. BMC Med Res Methodol.

[6] Nguyen Tran L (2017). Even it Up: How to tackle inequality in Vietnam.

[7] General Statistics Office of Vietnam (2018). Statistical summary book of Vietnam 2018.

[8] General Statistics Office of Vietnam (2019). Result of the Vietnam household living standards survey 2018.

[9] Weiss B, Dang M, Trung L, Nguyen MC, Thuy NTH, Pollack A (2014). A nationally representative epidemiological and risk factor assessment of child mental health in Vietnam. Int Perspect Psychol.

[10] Nguyen PVN, Hong TK, Hoang T, Robert A (2013). R: High prevalence of overweight among adolescents in Ho Chi Minh City. Vietnam BMC Public Health.

[11] Ngan HTD, Tuyen LD, Van Phu P, Nambiar S (2018). Childhood overweight and obesity amongst primary school children in Hai Phong City, Vietnam. Asia Pac J Clin Nutr.

[12] Hong TK, Dibley MJ, Sibbritt D, Binh PN, Trang NH, Hanh T (2007). T: Overweight and obesity are rapidly emerging among adolescents in Ho Chi Minh City, Vietnam, 2002–2004. Int J Pediatr Obes.

[13] Wang Y (2001). Cross-national comparison of childhood obesity: the epidemic and the relationship between obesity and socioeconomic status. Int J Epidemiol.

[14] Trang NH, Hong TK, Dibley MJ, Sibbritt D (2009). W: Factors associated with physical inactivity in adolescents in Ho Chi Minh City, Vietnam. Med Sci Sports Exerc.

[15] Nguyen PVN, Hong TK, Robert A (2016). R: Excessive screen viewing time by adolescents and body fatness in a developing country: Vietnam. Asia Pac J Clin Nutr.

[16] Van PhamHoan MD (2008). Vietnam recommended dietary allowances 2007. Asia Pac J Clin Nutr.

[17] Popkin BM, Gordon-Larsen P (2004). The nutrition transition: worldwide obesity dynamics and their determinants. Int J Obes.

[18] Thi HT, Simioni M, Thomas-Agnan C (2018). Assessing the nonlinearity of the calorie-income relationship: an estimation strategy–with new insights on nutritional transition in Vietnam. World Dev.

[19] Bobakova D, Hamrik Z, Badura P, Sigmundova D, Nalecz H, Kalman M (2015). Test–retest reliability of selected physical activity and sedentary behaviour HBSC items in the Czech Republic, Slovakia and Poland. Int J Public Health.

[20] Bosakova L, Kolarcik P, Bobakova D, Sulcova M, Van Dijk JP, Reijneveld SA, Geckova A (2016). M: Test–retest reliability of the scale of participation in organized activities among adolescents in the Czech Republic and Slovakia. Int J Public Health.

[21] Booth ML, Okely AD, Chey T, Bauman A (2001). The reliability and validity of the physical activity questions in the WHO health behaviour in schoolchildren (HBSC) survey: a population study. Br J Sports Med.

[22] Haugland S, Wold B (2001). Subjective health complaints in adolescence—reliability and validity of survey methods. J Adolesc.

[23] Holubčíková J, Kudláček M, Širůček J, MadarasováGecková A (2018). Test-retest reliability of selected HBSC items measuring problem behaviour among Slovak and Czech adolescents. Cent Eur J Public Health.

[24] Vereecken CA, Maes L (2003). A Belgian study on the reliability and relative validity of the health behaviour in school-aged children food-frequency questionnaire. Public Health Nutr.

[25] Roberts C, Freeman J, Samdal O, Schnohr CW, De Looze ME, Gabhainn SN, International HBSC Study Group (2009). The Health Behaviour in School-aged Children (HBSC) study: methodological developments and current tensions. Int J Public Health.

[26] Kaplan GA, Camacho T (1983). Perceived health and mortality: a nine-year follow-up of the human population laboratory cohort. Am J Epidemiol.

[27] Cantril H (1965). The pattern of human concern.

[28] Prochaska JJ, Sallis JF, Long B (2001). A physical activity screening measure for use with adolescents in primary care. Arch Pediatr Adolesc Med.

[29] Bucksch J, Sigmundova D, Hamrik Z, Troped PJ, Melkevik O, Ahluwalia N, Inchley J (2016). International trends in adolescent screen-time behaviors from 2002 to 2010. J Adolesc Health.

[30] Sigmund E, Sigmundová D, Badura P, Kalman M, Hamrik Z, Pavelka J (2015). Temporal trends in overweight and obesity, physical activity and screen time among Czech adolescents from 2002 to 2014: A national health behaviour in school-aged children study. Int J Environ Res Public Health.

[31] Koo TK, Li MY (2016). A guideline of selecting and reporting intraclass correlation coefficients for reliability research. J Chiropr Med.

[32] Inchley J, Currie DB, Budisavljevic S, Torsheim T, Jåstad A, Cosma A, Arnarsson ÁM (2020). Spotlight on adolescent health and well-being: Findings from the 2017/2018 Health Behaviour in School-Aged Children (HBSC) survey in Europe and Canada.

[33] Cohen J (1988). Statistical power analysis for behavioral sciences.

[34] Kohl HW, Fulton JE, Caspersen CJ (2000). Assessment of physical activity among children and adolescents: a review and synthesis. Prev Med.

[35] Lubans DR, Hesketh K, Cliff DP, Barnett LM, Salmon J, Dollman J, Hardy LL (2011). A systematic review of the validity and reliability of sedentary behaviour measures used with children and adolescents. Obes Rev.

[36] Levin KA, Currie C (2014). Reliability and validity of an adapted version of the Cantril Ladder for use with adolescent samples. Soc Indic Res.

[37] Muldoon JC, Levin K, van der Sluijs W, Currie C (2010). Validating mental well-being items of the Scottish health behaviour in school-aged children (HBSC) survey.

[38] Bacak V, Olafsdottir S (2017). Gender and validity of self-rated health in nineteen European countries. Scand J Public Health.

[39] Pavot W, Diener E (1993). Review of the satisfaction with life scale. Psychol Asses.

[40] Tran NTh (2001). Discovering the identity of Vietnamese culture.

[41] Weisz JR, Suwanlert S, Chaiyasit W, Weiss B, Achenbach TM, Eastman KL (1993). Behavioral and emotional problems among Thai and American adolescents: parent reports for ages 12–16. J Abnorm Psychol.

[42] Lansford JE, Malone PS, Dodge KA, Chang L, Chaudhary N, Tapanya S, Deater-Deckard K (2010). Children’s perceptions of maternal hostility as a mediator of the link between discipline and children’s adjustment in four countries. Int J Behav Develop.

[43] Ng K, Hämylä R, Tynjälä J, Villberg J, Tammelin T, Kannas L, Kokko S (2019). Test-retest reliability of adolescents’ self-reported physical activity item in two consecutive surveys. Archives of Public Health.

[44] Rangul V, Holmen TL, Kurtze N, Cuypers K, Midthjell K (2008). Reliability and validity of two frequently used self-administered physical activity questionnaires in adolescents. BMC Med Res Methodol.

[45] Lanfer A, Hebestreit A, Ahrens W, Krogh V, Sieri S, Lissner L, Pala V (2011). Reproducibility of food consumption frequencies derived from the children's eating habits questionnaire used in the IDEFICS study. Int J Obes.

[46] Vereecken CA, Rossi S, Giacchi MV, Maes L (2008). Comparison of a short food-frequency questionnaire and derived indices with a seven-day diet record in Belgian and Italian children. Int J Public Health.

[47] Wong JE, Parnell WR, Black KE, Skidmore PM (2012). Reliability and relative validity of a food frequency questionnaire to assess food group intakes in New Zealand adolescents. Nutr J.

[48] Speck BJ, Bradley CB, Harrell JS, Belyea MJ (2001). A food frequency questionnaire for youth: psychometric analysis and summary of eating habits in adolescents. J Adolesc Health.

[49] Moreno LA, Gottrand F, Huybrechts I, Ruiz JR, González-Gross M, DeHenauw S, HELENA Study Group (2014). Nutrition and lifestyle in european adolescents: the HELENA (Healthy Lifestyle in Europe by Nutrition in Adolescence) study. Adv Nutr..

[50] Asian Development Bank (2020). VIET NAM Secondary Education Sector Assessment, Strategy, and Road Map.

